# Ulnar Neuropathy at the Elbow Associated With Focal Demyelination in the Proximal Forearm and Intraoperative Imaging Correlation

**DOI:** 10.3389/fneur.2019.00292

**Published:** 2019-03-27

**Authors:** Ahmad R. Abuzinadah, Bassam M. J. Addas

**Affiliations:** ^1^Neurology Division, Internal Medicine Department, College of Medicine and King Abdulaziz University Hospital, King Abdulaziz University, Jeddah, Saudi Arabia; ^2^Neurosurgery Division, Surgery Department, King Abdul-Aziz University Hospital, Jeddah, Saudi Arabia

**Keywords:** ulnar, cubital tunnel, conduction block, entrapment, neuropathy

## Abstract

Ulnar nerve focal demyelination (FD) in the forearm [defined as conduction block (CB) and or temporal dispersion (TD)] has been described with immune-mediated neuropathy and with compression affecting the forearm segment of the nerve. The association of FD in the forearm with entrapment ulnar neuropathy at the elbow, as well as the intraoperative imaging of the abnormal ulnar nerve at the flexor carpi ulnaris muscle level (FCU), has not been reported before. We report a 33-years-old woman presented with only sensory symptoms of the right hand suggestive of right ulnar neuropathy for the last 10 years. On clinical examination, she had reduced pinprick sensation on the little and ring fingers with no motor deficit. Nerve-conduction study showed slowing of conduction velocity across the elbow on the right when recording at the abductor digiti minimi (ADM) and first dorsal interossei (FDI). There was 63% amplitude drop when stimulating below the elbow compared to distal stimulation at the wrist. Increment inching study localized the block at 5 cm distal to the medial epicondyle. During surgical transposition, the ulnar nerve was swollen, and edematous in the segment where the nerve enters the FCU muscle, which provides a physiological explanation for the electrophysiological findings. After the surgery, the patient reported complete resolution of the symptoms. This case demonstrate that ulnar nerve motor potential FD at the proximal forearm could be recorded and it is still compatible with ulnar-nerve entrapment at the elbow.

## Introduction

Ulnar neuropathy at the elbow is the second most common focal neuropathy of the upper limb (1). Electrodiagnostic studies are often requested in order to confirm diagnosis and to localize ulnar-nerve involvement at the elbow (1). The site of compression at the elbow occurs most commonly at the retroepicondylar (RTC) groove (76%), followed by entrapment between the two heads of flexor carpi ulnaris (FCU) under the humeroulnar aponeurotic arcade (HUA) (17%), also known as the cubital tunnel (2, 3). Entrapment at the arcade of Struthers is very rarely encountered (3). Ulnar-nerve entrapment at the elbow is diagnosed when motor-nerve potential conduction velocity across the elbow is <50 m/s, conduction velocity across the elbow is 10 m/s less than conduction velocity in the forearm, or a conduction block of >20% drop in compound motor action potential above the elbow compared to below elbow (4). The authors report a case of ulnar neuropathy at the elbow with a significant amplitude drop in the forearm segment due to focal demyelination, which was correlated with the intraoperative findings of an anatomically abnormal segment of the ulnar nerve at the level of the FCU muscle.

## Case Presentation

A 33-years-old woman, previously healthy, presented with 10 years' history of tingling in the little and ring fingers and over the hypothenar part of the palm on the right side. The tingling has progressed from waking her up every night to preventing her from sleep. She had no hand-grip weakness or difficulty with hand dexterity. On examination, she had reduced pinprick sensation in the ulnar distribution of the right hand compared to the normal side of the right hand as well as the left-hand ulnar distribution. Motor examination was normal. The patient had a nerve-conduction study performed at an outside hospital that was suggestive of ulnar-nerve entrapment at the wrist level.

**Figure 1:**
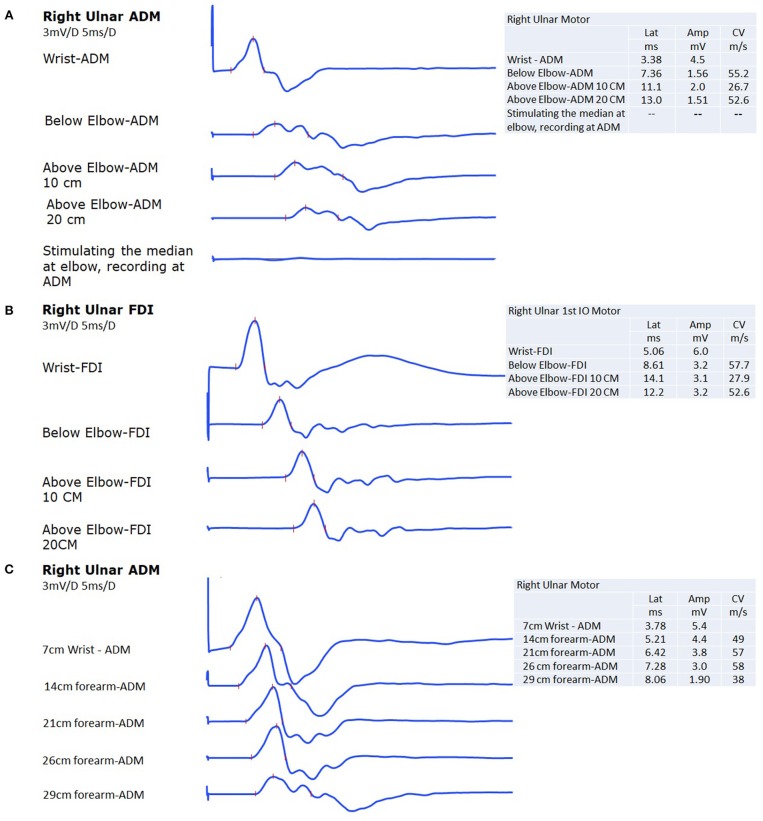
**(A)** The ulnar nerve compound motor action potential at the abductor digiti minimi (ADM) shows 65% drop in amplitude with 30% drop in the area and 63% prolongation in the duration when the nerve is stimulated below the elbow as compared to stimulation at the wrist. There was a slowing in conduction velocity across the elbow. The last trace, shows no motor response from ADM when stimulating the median nerve at the elbow. **(B)** The ulnar nerve compound motor action potential at the first dorsal interossei (FDI) shows 47% drop in amplitude with 53% drop in the area and 17% prolongation in the duration when the nerve is stimulated below the elbow as compared to stimulation at the wrist. There was a slowing in conduction velocity across the elbow. **(C)** The last area where the amplitude was partially maintained before the 63% amplitude drop occurred (when recording from ADM) 6 cm below the elbow, indicating that the maximum area of focal demyelination occurs just proximal to that point at around 5 cm below the elbow.

The nerve conduction study showed normal median motor and sensory action potentials. Ulnar-nerve conduction showed reduced ulnar-nerve sensory action potential at the fifth digit. The ulnar motor conduction amplitude when distally stimulating the ulnar nerve and recording at the abductor digiti minimi at the wrist was normal; however, there was a 63% amplitude drop when stimulating below the elbow compared to distal stimulation at the wrist (Figure 1A). This 63% amplitude drop was associated with 30% drop in the area and 63% prolongation in the duration. Conduction velocity across the elbow was slow (26 m/s) compared to the forearm segment (55 m/s) or from above the elbow to the axilla segment (52 m/s). There was no motor response from ADM when stimulating the median nerve at the elbow (Figure 1A). In addition, ulnar motor conduction amplitude when stimulated below the elbow and recorded at the first dorsal interossei showed a 47% amplitude drop when compared to distal stimulation at the wrist (Figure 1B). This 47% amplitude drop was associated with 53% drop in the area and 17% prolongation in the duration. Conduction velocity across the elbow was slow (27 m/s) compared to the forearm segment (57 m/s) or from above the elbow to the axilla segment (52 m/s). The left side ulnar motor potentials were normal with no slowing of conduction velocity across the elbow.

In order to rule out pathology in the forearm on the right that might explain the conduction block, we traced the right ulnar nerve motor potential up to the elbow (Figure 1C). The major part of the amplitude drop (37%), as well as conduction velocity slowing, occurred between 3 and 6 cm distal to the medial epicondyle. There was loss of amplitude as the nerve descended in the forearm, but this is likely attributed to a deeper course distally.

**Figure 2:**
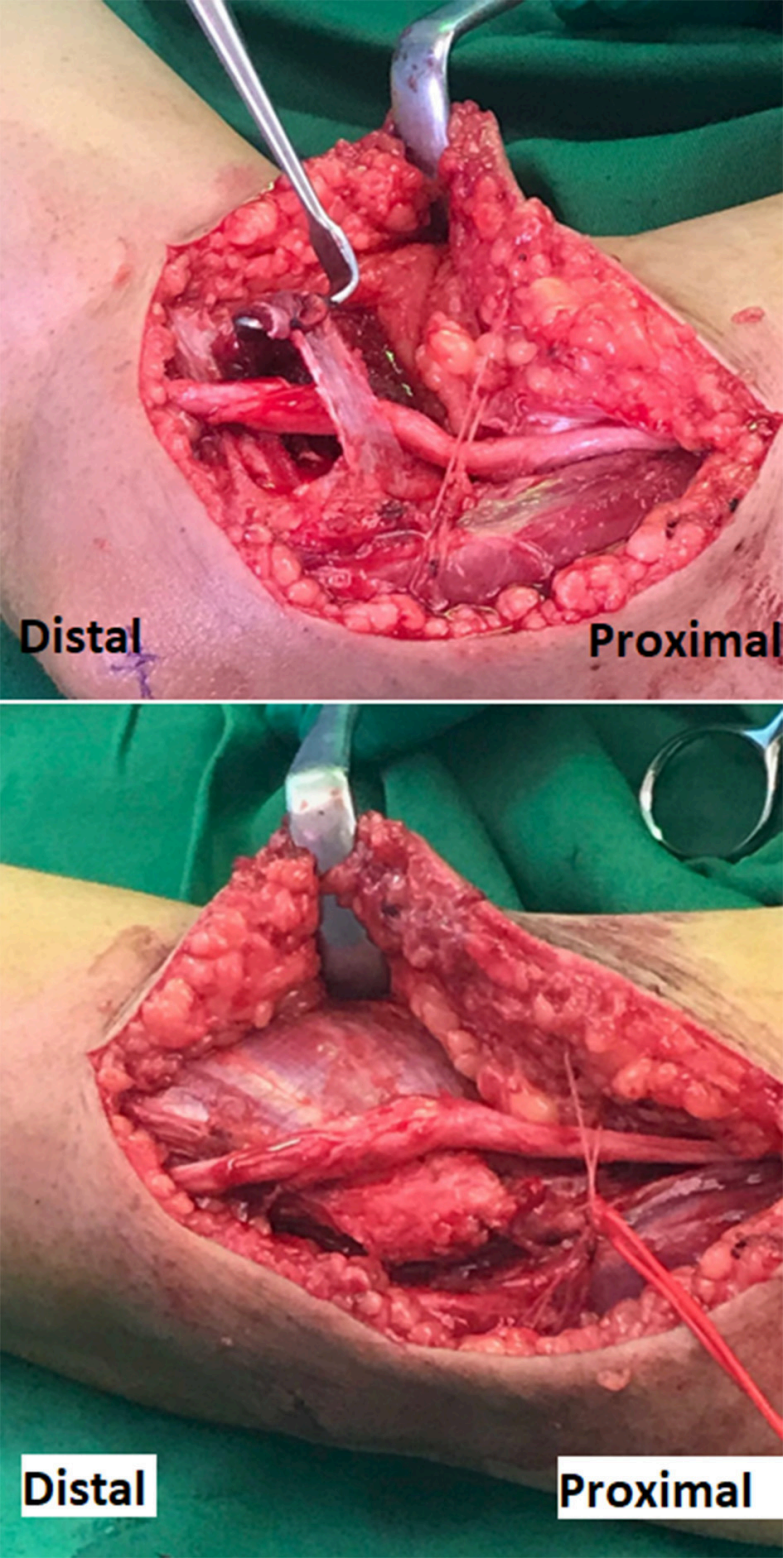
Surgical photograph of the right elbow demonstrating the released ulnar nerve from the cubital tunnel placed over the flexor muscle mass, in preparation to complete the transposition under a fascial sling. The edematous segment of the ulnar nerve shown is the segment that normally runs between the two heads of FCU muscle. The red vessel loop encircles the posterior branches of the medial antebrachial cutaneous nerve that crosses the surgical incision.

Surgical transposition was conducted and showed nerve swelling distal to the medial epicondyle between the two heads of FCU (Figure 2). The swollen segment of the nerve extended for additional 2 cm distal to the two heads of FCU and the HUA with no additional cause of entrapment was identified at that segment during the surgical exploration (Figure 2). Follow up at 6 months after the surgery, the patient has reported complete resolution of the symptoms including absence of tingling sensation and normal sleep.

## Discussion

Our case demonstrates that a focal demyelination in the forearm could occur in a case of entrapment ulnar neuropathy at the elbow at the cubital tunnel. Surgeons regularly check for entrapment sites, but our case documented an imaging picture of entrapment between the two heads of FCU (known as cubital-tunnel syndrome) for the first time. The importance of recognizing this unusual site of the focal demyelination and particularly conduction block is to avoid misdiagnosis as immune-mediated neuropathy and to avoid mislocalizing the entrapment site, which may lead to a wrong surgical release site. The expected site of the focal demyelination and conduction block with entrapment ulnar neuropathy at the elbow is seen when comparing motor potential amplitude above and below the elbow (5, 6).

Focal demyelination (e.g., conduction block) in the forearm segment of the ulnar nerve (non-entrapment site) is usually caused by syndromes other than ulnar entrapment at the elbow (7). It is usually considered as a sign of immune-mediated demyelinating neuropathy, and commonly associated with Lewis–Sumner syndrome (LSS) (7). Conduction block in the forearm occurs in 59% of patients with LSS, and that includes the ulnar nerve in 53% of the patients. Immune-mediated therapy in this condition results in improvement in 73% of cases (7). The fibrovascular band coursing from the ulnar artery to the FCU muscle may also cause ulnar neuropathy with conduction block in the distal forearm (8, 9). However, the site of the conduction block in this conduction is expected to occur at 7 cm proximal to the ulnar styloid (8). Distal ulnar neuropathy could also occur with acute calcific tendinitis (10). Ulnar-nerve conduction block in the forearm can be seen in cases of Martin-Gruber anastomosis; however, the absence of motor response from ADM when stimulating the median nerve at the elbow suggests that Martin-Gruber anastomosis does not exist in the patient (3).

In our case, there was a drop in the ulnar nerve motor potential amplitude across the forearm segment. The maximum area of focal demyelination occurs at 5 cm below the elbow (Figure 1C). When applying the American Academy of Neuromuscular and Electrodiagnostic Medicine (AANEM) consensus guidelines, this focal demyelination was mainly due to temporal dispersion when ulnar nerve motor potential was recorded from ADM as the prolongation in the duration of the motor potential exceeded 60% with only 30% drop in area (11). However, this focal demyelination was mainly due to definite partial conduction block when ulnar nerve motor potential was recorded from FDI as there was 62% drop in the area with only 17% prolongation in the duration (11). The clue that the site of entrapment was at the elbow is the presence of clearly slow conduction velocity across the elbow. Velocity became faster when measured from the axilla down to above the elbow localization, and this finding was replicated by stimulating the FDI muscle. As the nerve ascended in the forearm, it goes deeper; however, it is expected to lose some amplitude throughout that course due to technical reasons (3). However, usually within 2–3 cm from the medial epicondyle, the amplitude becomes similar to the amplitude with stimulation at the wrist (3). During surgical release and transposition, the nerve appeared swollen and edematous for 2–3 cm of the distal to medial epicondyle and the swollen segment extended for additional 2–3 cm distal to the two heads of FCU and the HUA, which provides a physiological explanation for the focal demyelination and conduction block. Complete resolution of symptoms following transposition surgery provides a correlation between the symptoms and electrodiagnostic findings.

High-resolution sonography of the ulnar nerve is emerging as a new diagnostic tool for ulnar neuropathy at the elbow (UNE) (12). It has similar diagnostic sensitivity for UNE as the electrodiagnostic studies (78%), however, the combination of both improves the sensitivity to 98% (13). Another study showed that the sonographic localizing utility (72%) was similar to that of electrodiagnostic studies (71%), however, the combination of both studies improves the localizing utility to 91% (2). In our case, the electrodiagnostic study was sufficient to localize the site of entrapment (13). Sonography may have the advantage of identifying certain etiologies such as leprosy or nerve sheath tumors through certain sonographic features (14, 15). Nerve enlargement as evident by increased ulnar nerve diameter or increased the cross-sectional area are the usual sonographic signs of UNE (2, 13). The intraoperative imaging of swollen ulnar nerve at the elbow provides a clinical basis for these usual sonographic findings.

## Data Availability

All datasets generated for this study are included in the manuscript and/or the supplementary files.

## Consent

A written informed consent was obtained from the participant for the publication of this case report.

## Author Contributions

AA: writing the manuscript and the electrodiagnostic findings. BA: editing the manuscript and writing the surgical findings.

### Conflict of Interest Statement

The authors declare that the research was conducted in the absence of any commercial or financial relationships that could be construed as a potential conflict of interest.
